# Safety and effectiveness of double microcatheter technique in the treatment of ruptured aneurysms of anterior cerebral circulation

**DOI:** 10.3389/fneur.2022.1015304

**Published:** 2022-12-05

**Authors:** Xintong Zhao, Zihuan Zhang, Jiaqiang Liu, Feiyun Qin, Liying Hu, Zhenbao Li

**Affiliations:** Department of Neurosurgery, The First Affiliated Hospital of Wannan Medical College, Wuhu, China

**Keywords:** double microcatheter technique, stent-assisted coiling, balloon-assisted coiling, ruptured aneurysm, anterior cerebral circulation

## Abstract

**Objective:**

To evaluate the safety and effectiveness of the double microcatheter technique in the treatment of ruptured aneurysms of the anterior cerebral circulation.

**Methods:**

Between 2012 and 2019, 113 patients with ruptured aneurysms of the anterior cerebral circulation were treated using the double microcatheter technique. Clinical records, angiographic results, and procedure-related complications were reviewed. Clinical and angiographic follow-up was performed.

**Results:**

Complete occlusion, neck remnant, and partial occlusion were, respectively, recorded in 56.6, 38.9, and 4.4% of the total cases. For all patients, the incidence of intraoperative complications was 5.3% (6/113), and the overall rate of morbidity was 10.6% (12/113). Before discharge, three patients (2.7%) died. There was no procedure-related mortality. At discharge, favorable outcomes were observed in 79.6% (90/113) of the patients. High Hunt-Hess grades and receiving a craniotomy or external ventricular drainage were risk factors for clinical outcomes at discharge. Clinical follow-up was performed in 91 patients at a mean interval of 14.07 ± 11.68 months. At follow-up, favorable outcomes were observed in 92.3% (84/91) of the patients. Angiographic follow-up was performed in 66 patients at an average of 11.53 ± 11.13 months. The recurrence rate was 37.9%. Of these patients, 13 (19.7%) received retreatment.

**Conclusion:**

The double microcatheter technique can be performed in ruptured aneurysms with high technical success and low morbidity/mortality. However, recurrence remains a problem, and patients should be followed up regularly.

## Introduction

Since the publication of the International Subarachnoid Aneurysm Trial (ISAT) in 2002 (1), endovascular approaches have become the first choice for the treatment of intracranial aneurysms. Along with the advances in operative devices and techniques, the prognosis of ruptured aneurysms has been improved significantly. However, wide-necked and irregular aneurysms still represent a significant challenge. Stent- or balloon-assisted coiling and flow diversion can be used in these cases (2–4). However, the deployment of a stent in ruptured aneurysms may increase procedure-related complications, such as rebleeding and thrombosis (5–7). Balloon-assisted coiling requires a temporary blockage of blood flow and may also incur an increased risk of related complications (8, 9). In addition, navigating the stent- or balloon-delivery system becomes difficult in small or tortuous vessels.

Baxter et al. (10) reported the double microcatheter technique in 1998. The advantages of this approach are that there is no blockage of blood flow and that it does not recommend antiplatelet medications. Thus, the double microcatheter technique represents an alternative method to treat wide-necked and irregular aneurysms. Although some recent studies reported on the use of the double microcatheter technique (11–13), reporting on its use for the treatment of ruptured aneurysms of the anterior cerebral circulation is rarer. Here, we report the safety and effectiveness of the double microcatheter technique in anterior circulation ruptured aneurysms.

## Materials and methods

### Patient selection

From September 2012 to November 2019, a total of 113 patients with ruptured aneurysms of the anterior cerebral circulation were admitted to our hospital and treated with the double microcatheter technique. All patients had given their informed consent to participate. Cases of dissecting and fusiform aneurysms were excluded. Subarachnoid hemorrhage was confirmed by CT scanning. The clinical condition at admission was classified by the Hunt-Hess grade. Most patients received CT angiography (CTA), which was confirmed by digital subtraction angiography (DSA). Wide-necked aneurysms were defined as neck diameter ≥4 mm or dome-to-neck ratio <2. All procedures were performed within 48 h of being admitted to the hospital.

### Endovascular procedure

All procedures were performed under general anesthesia and systemic heparinization. A 6-F guiding catheter was placed into the internal carotid artery through the right femoral artery. Frontal, lateral, and 3D angiography were performed in all patients to analyze the size and configuration of the aneurysms. The treatment strategy was determined by the operators according to the diameter of the parent artery and the characteristics of the aneurysm. Before the procedure, we verified that the guiding catheter and the parent artery could accommodate two microcatheters. Echelon-10 or Headway-17 microcatheters were mostly used during the procedures. The double microcatheter technique is usually used in wide-necked, irregular, or branch-incorporated aneurysms. Two different strategies (i.e., coil “interleaving” and “locking” techniques) were described by Durst et al. (12).

During the procedure, two microcatheters were navigated into the aneurysm under roadmap guidance after the selection of working projections. The two microcatheters should be preshaped with different tip curves to optimally reach different portions of the aneurysm. After the first microcatheter was in place, the first coil was fully or partially filled into the aneurysm. As the advancement of the second microcatheter may cause the first one to migrate, the tension of the first microcatheter should be adjusted as appropriate. Still, the possibility of rebleeding was low due to the buffering effect of the first coil, even if the tension of the first microcatheter changed.

**Figure 1:**
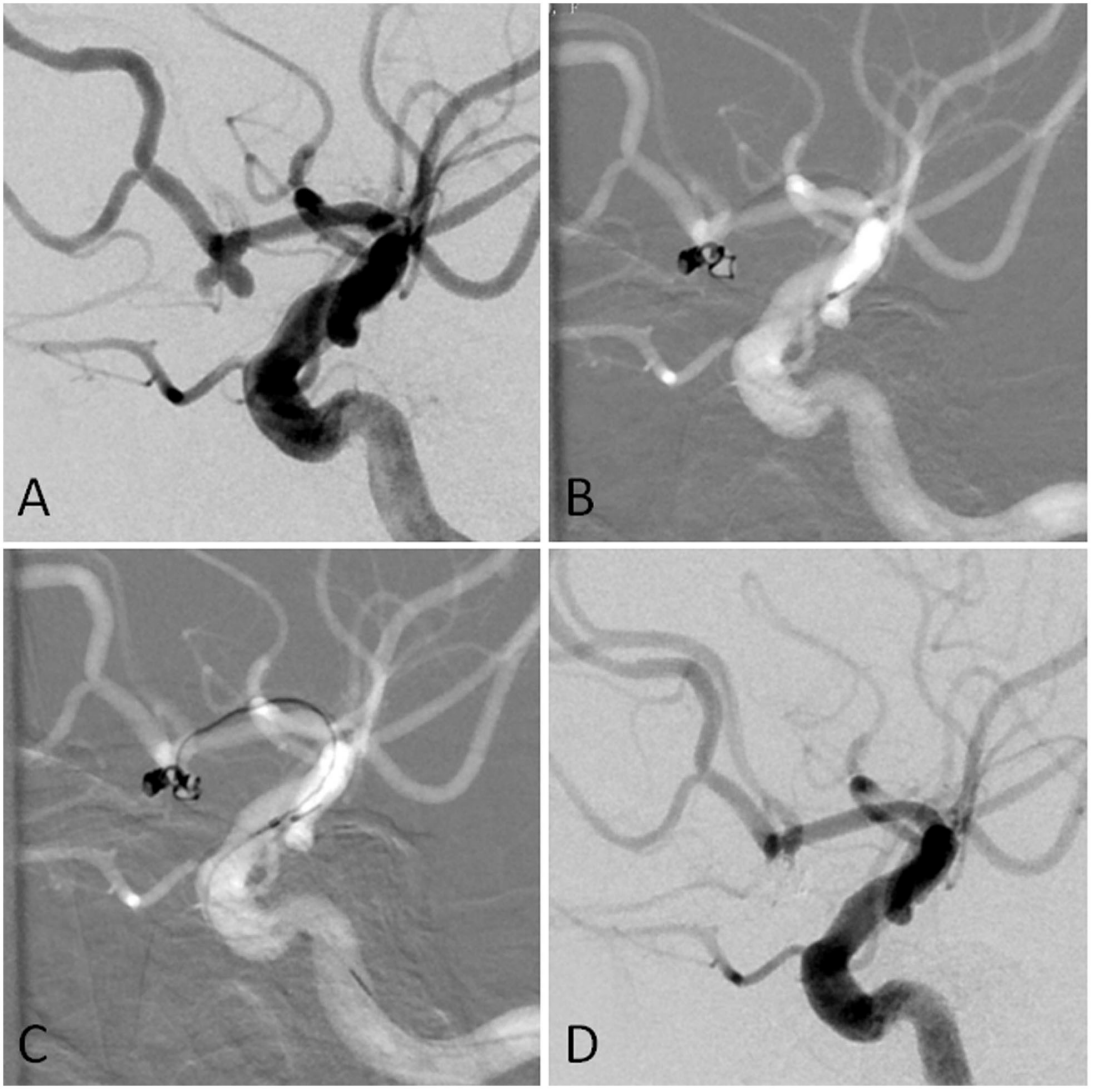
**(A)** The angiographic imaging showed AcomA aneurysm with the daughter sac. **(B)** The first was navigated into the left sac of the aneurysm. The first coil was fully advanced into the aneurysm. Part of the first coil and the microcatheter entered into the right sac in the end. **(C)** Another microcatheter was navigated into the aneurysm. **(D)** The aneurysm was completely occluded.

**Figure 2:**
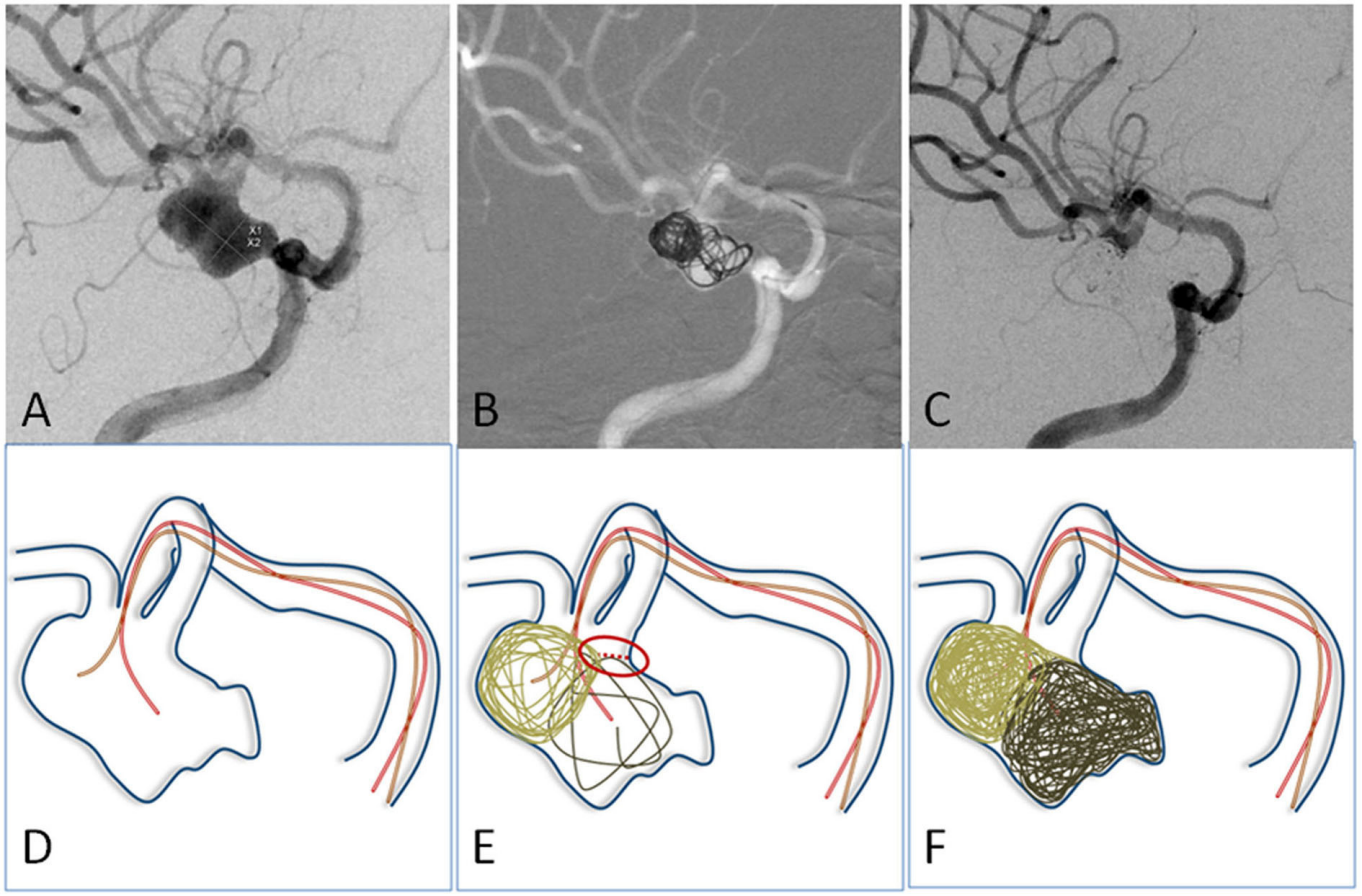
**(A)** The angiographic imaging showed an MCA aneurysm with lateral growth. **(D)** Illustration: Two microcatheters with different curves were navigated into different portions of the aneurysm. **(B,E)** The first two coils were advanced into aneurysms through two microcatheters to form a stable frame. They supported and embraced each other. **(C,F)** The aneurysm was embolized with neck remodeling.

**Figure 3:**
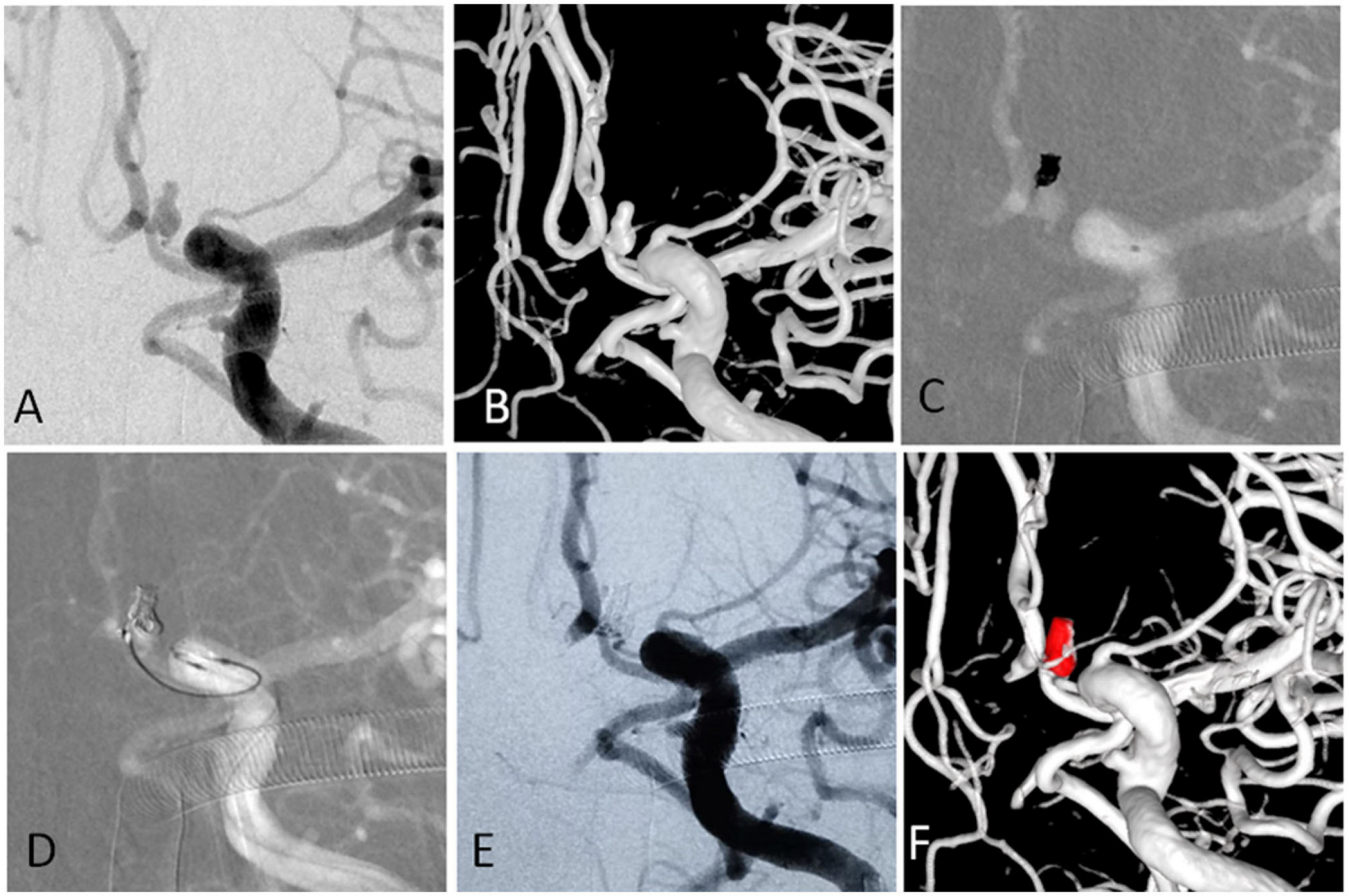
**(A,B)** The angiographic and 3-D imaging showed an anterior communicating artery complex aneurysm. Heubner artery originated from the neck. **(C)** The first microcatheter was navigated into the aneurysm, and the first coil was fully advanced into the aneurysm. **(D)** The second microcatheter was navigated into the aneurysm to protect the Heubner artery from being occluded. **(E,F)** The aneurysm was completely occluded, and the Heubner artery was patent.

For aneurysms with a large daughter sac (Figure 1), usually one microcatheter is navigated into the daughter sac if there was no difficulty. After the first coil was fully or partially deployed, the second coil was advanced into the aneurysm through the second microcatheter, and the two coils intertwined with each other to form a stable frame. One of the coils was then detached, while the other coil remained attached to the pusher wire. Coils were then introduced through the second catheter until the aneurysm was packed as densely as possible. If blood flow was affected during this procedure, one microcatheter was removed once a stable frame was formed. For wide-necked aneurysms with lateral growth, the double-parallel framing coil technique was first attempted. To this end, two microcatheters with different shapes were consecutively or parallelly advanced into the aneurysm so that the two framing coils form a stable structure. Then, the procedure continued as described earlier. An illustrative case is presented in Figure 2. For branch-incorporated aneurysms (Figure 3), one microcatheter was placed at the orifice of the branch to prevent its occlusion, and a second microcatheter was then used to coil the aneurysm.

In two cases with wide-necked aneurysms planned for stent-assisted coiling, the first coil formed a good frame, but this was not stable enough. Since the coil may herniate into the parent artery if it became detached, the stent-delivery catheter was removed. The other microcatheter was then navigated into the aneurysm, and a second coil was deployed to stabilize the frame.

In severe cases with a Hunt-Hess score of IV–V, ventricular drainage is usually performed first, followed by endovascular coiling, because relieving intracranial pressure is even more critical. For cases with a Hunt-Hess of III or below and with hydrocephalus after coiling, endovascular coiling is usually performed first, followed by EVD.

### Clinical and angiographic assessment

The clinical outcomes at discharge and at the last follow-up were scored using the Glasgow Outcome Scale (GOS). Patients who attained a GOS score of 4–5 were defined as having a favorable outcome. Patients with a GOS score of 1–3 were defined as having a poor outcome. At discharge, all patients were recommended to undergo DSA at 3, 9, and 21 months after the procedure. If the patient has no recurrence after 21 months, the follow-up interval would be extended. The immediate angiographic outcomes at the end of the procedure were classified as complete obliteration, neck remnant, and partial occlusion (contrast filling in the aneurysm sac). Angiographic outcomes at follow-up were classified as stable (no change in coil configuration, obliteration grade, or contrast filling), improved (progressive occlusion or involution of the neck remnant or contrast filling in an aneurysm), and recanalized (aneurysm recurrence evident due to neck growth, coil compaction, coil extrusion by aneurysm degradation, or new sac formation) (14).

### Statistical analysis

Statistical analysis was performed using SPSS software, version 19.0 (IBM Inc., Chicago, IL, USA). Univariable and multivariable logistic regression were used to analyze the risk factors for procedure-related morbidities and clinical outcomes at discharge. A multivariable proportional hazards regression (Cox) model was used to analyze the risk factors for clinical and angiographic outcomes at follow-up. A *P* < 0.05 was considered to be statistically significant.

## Results

### Patient and aneurysm characteristics

Out of the 113 patients, 26 were men and 87 were women. The mean age was 60.12 ± 10.51 years (range, 31–82 years). The Hunt-Hess grade at admission was I in 18 patients, II in 42 patients, III in 36 patients, IV in 12 patients, and V in 5 patients. There were 40 cases of hypertension and 4 of diabetes. Seven patients had both hypertension and diabetes. Pre-procedure CT scanning showed that 14 cases had an intracranial hematoma (without brain herniation), 9 had hydrocephalus, and 4 had both.

The double microcatheter technique was used to treat one patient with bilateral posterior communicating artery aneurysms and 112 single aneurysms in the remaining 112 patients. There were 36 anterior communicating artery aneurysms, 59 posterior communicating artery aneurysms, 12 middle cerebral artery aneurysms, 3 aneurysms of an ophthalmic segment of the internal carotid artery, 3 anterior choroidal artery aneurysms, and one aneurysm at the A1 segment of the anterior cerebral artery. Of all aneurysms, 89 (78.1%) were wide-necked and 61 (53.5%) presented with a daughter sac or irregular configuration. The neck diameter ranged from 1.6 to 9 mm, with an average of 3.60 ± 1.12 mm. Maximum diameter was on average 6.30 ± 2.50 mm (range, 2.9–13 mm). The dome/neck ratio ranged from 0.7 to 3.92 with an average of 1.80 ± 0.59.

### Perioperative complications

Throughout all the procedures, intraoperative bleeding occurred in 2 out of 113 patients (including a patient with rebleeding during anesthesia). In addition, thrombosis or slow blood flow through the parent artery was recorded in 4 cases. Once the aneurysm was packed densely, tirofiban was infused through the microcatheter until the thrombus disappeared and blood flow returned to normal. No postoperative neurological deficits were observed in any patient. The incidence of intraoperative complications was 5.3% (6/113). One patient suffered from increased bleeding and ischemic events simultaneously after the procedure, and he received a decompressive craniotomy. After the procedure, five patients suffered from ischemic events, and one of them recovered without neurological deficits after the administration of anti-thrombotic medication. The overall rate of morbidity was 10.6% (12 out of 113). Because of the low morbidity rate, there was no significant correlation between patients or aneurysm characteristics and morbidity.

Additionally, six patients underwent external ventricular drainage (EVD) for hydrocephalus. Due to hydrocephalus, hematoma, or severe vasospasm, craniotomy was performed in seven patients and craniotomy with EVD in three patients. Among the patients receiving EVD and/or craniotomy, seven had anterior communicating artery aneurysms, eight had posterior communicating artery aneurysms, and one had a middle cerebral artery aneurysm. Ventriculoperitoneal shunts were performed in six patients due to delayed hydrocephalus. An IVC filter was implanted in one patient due to deep venous thrombosis.

### Clinical outcomes

In this series of patients, the clinical outcomes at discharge were as follows: a GOS score of 1 in three patients, a GOS score of 2 in three patients, a GOS score of 3 in 17 patients, a GOS score of 4 in 14 patients, and a GOS score of 5 in 76 patients. The mortality rate at discharge was 2.7% (3/113). There was no procedure-related mortality. Of the three patients who died before discharge, there were two patients with a Hunt-Hess grade of V and one patient with a Hunt-Hess grade of IV at admission. Favorable outcomes at discharge were observed in 79.6% (90/113) of patients. Table 1 shows the risk factors for clinical outcomes at discharge, based on univariable and multivariable logistic regression. High Hunt-Hess grade and receiving craniotomy or EVD were risk factors for poor clinical outcomes at discharge.

**Table 1:** Univariable and multivariable logistic analyses of risk factors for poor outcomes at discharge.

**Factors**	**Univariable**		**Multivariable**	
	**OR (95%CI)**	***P***	**OR (95%CI)**	***P***
Hunt-Hess grade	0.036 (0.010–0.131)	0.000	0.050 (0.008–0.328)	0.002
Craniotomy or EVD	0.027 (0.006–0.109)	0.000	0.050 (0.006–0.425)	0.006
Age	2.186 (0.821–5.823)	0.118	4.445 (0.820–24.094)	0.084
Hematoma	0.136 (0.048–0.389)	0.000	0.454 (0.088–2.353)	0.347
Hydrocephalus	0.110 (0.032–0.383)	0.001	0.668 (0.095–4.702)	0.685
Wide neck	0.692 (0.212–2.259)	0.542		
Packing density	0.713 (0.310–1.638)	0.425		

EVD, external ventricular drainage.

Apart from three patients dying before discharge and 19 patients lost to follow-up, clinical follow-up was available in 91 patients at a mean interval of 14.07 ± 11.68 months (range, 3–71 months). Among them, seven patients had a GOS score of 3, six patients had a GOS score of 4, and 78 patients had a GOS score of 5. None of these patients suffered from rebleeding. Favorable outcomes were obtained in 92.3% (84/91) of patients. Factors influencing outcome in the multivariable proportional hazards model are shown in Table 2. Among the variables considered, a high Hunt-Hess grade was the only risk factor for clinical outcomes at follow-up.

**Table 2:** Multivariable proportional hazards model analysis for poor outcomes at follow-up.

**Factors**	**Hazard ratio**	**(95%CI)**	***P***
Age	76.489	0.465–12,592.967	0.096
Hunt-Hess grade	56.072	2.655–1,184.384	0.010
Hypertension	0.042	0.001–1.381	0.075
Hematoma	0.109	0.003–4.362	0.239
Hydrocephalus	0.642	0.024–17.008	0.791
Craniotomy or EVD	6.166	0.142–267.320	0.344

EVD, external ventricular drainage.

### Angiographic outcomes

Postoperative angiography demonstrated complete occlusion in 64 (56.1%), neck remnant in 44 (38.6%), and partial occlusion in six (5.3%) aneurysms. Daughter sac coiling was performed on two patients.

Angiographic follow-up was performed in 66 patients at an average of 11.53 ± 11.13 months (range: 3–71 months). In 41 aneurysms, the occlusion was stable or improved. Recurrence was found in 25 aneurysms (37.9%), and 13 of these (19.7%) received retreatment.

## Discussion

Endovascular therapy has become the first choice of treatment for intracranial aneurysms. Despite its high success rate, the treatment of wide-necked and irregular aneurysms of the anterior cerebral circulation is still a challenge because of coil instability and a high recurrence rate (15). The improvement of devices and techniques expanded the usage of endovascular coiling techniques such as stent- or balloon-assisted coiling, the double microcatheter technique, and flow diversion (2–4, 12) for the treatment of wide-necked or irregular aneurysms. However, the adequacy of stent- or balloon-assisted coiling for ruptured aneurysms in the acute stage is still debated.

Stent-assisted coiling is commonly used in the treatment of unruptured, wide-necked aneurysms. The deployment of a stent prevents the coil from protruding into the parent artery and may thus reduce the recurrence rate (16, 17). However, the deployment of a stent in the acute stage may incur additional complications, such as in-stent thrombosis (7, 18, 19). Fan et al. (18) reported that thrombosis occurred in 15.9% of the patients after stent-assisted coiling vs. 3.8% of the patients after coiling alone for ruptured anterior communicating artery aneurysms. However, postoperative complications did not significantly differ between the two groups. In turn, the results of a meta-analysis of 10 retrospective cohort studies performed by Hong et al. (20) demonstrated comparable, non-significant differences in the all-complication rate associated with stent-assisted coiling (17.6%) and conventional coiling (15.9%). However, routine usage of antiplatelet medications before stent-assisted coil embolization may increase the rate of rebleeding, especially in patients who require EVD, craniotomy, or ventriculoperitoneal shunt (7, 21, 22). Additionally, it is difficult to navigate the stent-delivery catheter through tortuous and small vessels. There are several recent reports of the flow diversion treatment for intracranial aneurysms (23). However, with this technique, the occlusion of aneurysms may require weeks or months, and rebleeding is not excluded (24).

The balloon remodeling technique, described by Moret et al. (25), provided a novel approach for the treatment of wide-necked or irregular aneurysms. Once deployed into the aneurysm, the balloon is inflated, which temporarily blocks blood flow, protects the aneurysm neck, decreases the risk of rupture, and increases coil packing density. In addition, this approach does not require the recommendation of antiplatelet medications perioperatively. However, delayed coil migration (26), as well as bleeding and thrombosis events (9, 27), have been reported as potential complications of this technique. Sluzewski et al. (9) reported that procedure-related complications were higher for balloon-assisted coiling (14.1%) compared to coiling alone (3%). Additionally, as with stent-assisted coiling, navigation of the balloon catheter into tortuous and narrow vessels is often difficult. Therefore, the use of balloon-assisted coiling is not supported by some authors (9, 27).

Since the introduction by Baxter et al. (10) of the double microcatheter technique in 1998, several studies addressed its effectiveness in the treatment of intracranial aneurysms (11–13). The advantages of the double microcatheter technique are that there is no blockage of the blood flow or that antiplatelet medications are not recommended. The procedure is relatively simple compared with stent- or balloon-assisted coiling when it is used in wide-necked or irregular aneurysms. The tips of the two microcatheters must have different curves to reach different portions of the aneurysm and form a stable frame. However, as the advancement of the second microcatheter may cause the first one to migrate forward, care must be taken to properly adjust the tension of the first microcatheter. Durst et al. (12) reported three intraprocedural ruptures (3%) in 100 consecutive patients who underwent coil embolization of a wide-necked aneurysm using a dual microcatheter technique. In two of these cases, early identification of the rupture allowed successful coiling. In the remaining case, a rupture occurred because the microcatheter perforated the aneurysm dome, which led to bleeding, hydrocephalus, and patient death. In our cases, the first coil was usually advanced fully or partially into the aneurysm and remained attached before the advancement of the second microcatheter. Intraoperative bleeding occurred in two patients (including a patient with rebleeding during anesthesia). When rupture occurs, rebleeding may be controlled by rapid packing, neutralizing heparin, or lowering blood pressure. If these strategies did not work, we attempted to block the blood flow with a balloon or by compressing the internal carotid artery.

The double microcatheter technique can be used in a variety of aneurysms. First, it can be used in wide-necked aneurysms. To form a stable frame, two differently shaped microcatheters are navigated into different portions of aneurysms, as reported in several studies (11, 12). For wide-necked aneurysms with creeping growth, we first used the double parallel framing coils technique. Second, it can be used in aneurysms with a daughter sac. Some aneurysms have relatively large sacs, even larger than the aneurysm itself. The aneurysm and its daughter sac need to be coiled simultaneously in order to reduce the rate of rebleeding. If it is not difficult to insert, one microcatheter can be navigated into the daughter sac and the other one is navigated into the aneurysm sac. The daughter sac is usually the bleeding point. It can decrease the risk of rebleeding if the daughter sac can be packed densely. Kim et al. (28) reported one case of a fusiform aneurysm in the supraclinoid segment with a daughter sac. The patient received selective coiling of the daughter sac. Third, it can be used in branch-incorporated aneurysms. In order to protect the orifice of the branch from being occluded, one microcatheter was placed. Another microcatheter was used to coil the aneurysm. Kim et al. (28) reported this technique in 2018. Fourth, it can be used in elongated aneurysms (29). One microcatheter is navigated deeply into the aneurysm, and the other one is close to the neck. Additionally, it can be used in parent artery occlusion for ruptured vertebral artery aneurysms by bilateral vertebral artery approach. However, most vertebral artery aneurysms are dissecting ones. In this series of cases, the ruptured aneurysms in posterior cerebral circulation were excluded. The double microcatheter technique is associated with lower procedure-related complications compared to stent-assisted coiling (13). However, the recurrence rate may be higher. Procedure-related complications were reported to occur in ~20% of ruptured aneurysm patients receiving stent-assisted coiling (22, 30). However, for ruptured aneurysms treated by double microcatheter, the corresponding data are scarce. Yoon et al. (11) reported 56 patients with acutely ruptured wide-necked intracranial aneurysms treated with the double microcatheter technique. Procedure-related complications and permanent complication rates were 12.5 and 1.8%, respectively. Recurrence and retreatment occurred in 21 patients (56.8%) and 5 patients (13.5%), respectively. Favorable outcome at discharge was recorded in 36 (64.3%) patients. In our series of patients, the procedure-related complication rate was 5.3%, whereas recurrence and retreatment rates were 37.9 and 19.7%, respectively.

Favorable outcomes were obtained in 79.6% (90/113) of study patients at discharge. High Hunt-Hess grade and receiving EVD and/or craniotomy were risk factors for clinical outcome at discharge. Some authors reported that there was no significant difference in recurrence rate among patients treated with the double microcatheter technique or with stent- or balloon-assisted coiling (13, 31). However, more recurrence cases may arise over extended follow-up. Upon recurrence, retreatment may be chosen in cases with fewer morbidities after addressing potential complications and the risk of recurrence.

To the best of our knowledge, this is one of the largest studies describing the use of the double microcatheter technique. There are still some limitations to this study. Because this is a retrospective and non-randomized study, there is inevitable selection bias. Additionally, the number of patients was limited. Some patients were lost to follow-up because of changes in contact information. Some patients having poor outcomes were reluctant to follow up, especially with imaging follow-up such as DSA.

## Conclusion

Based on our experience, we conclude that the double microcatheter technique is a safe and effective treatment modality for ruptured aneurysms located in the anterior cerebral circulation. However, recurrence remains a problem, and patients should be followed up regularly. A randomized controlled trial with a larger sample would be informative to further analyze the safety and effectiveness of the double microcatheter technique.

## Data availability statement

The raw data supporting the conclusions of this article will be made available by the authors, without undue reservation.

## Author contributions

ZL: conception and design of the manuscript. XZ: writing the original draft, reviewing, and editing. ZZ, JL, FQ, and LH: data acquisition, data analysis, and reviewed the manuscript. All authors contributed to the article and approved the submitted version.

## Conflict of interest

The authors declare that the research was conducted in the absence of any commercial or financial relationships that could be construed as a potential conflict of interest.

## Publisher's note

All claims expressed in this article are solely those of the authors and do not necessarily represent those of their affiliated organizations, or those of the publisher, the editors and the reviewers. Any product that may be evaluated in this article, or claim that may be made by its manufacturer, is not guaranteed or endorsed by the publisher.
